# Outcomes of adult acquired buried penis (AABP) reconstruction: a multicentre cohort study

**DOI:** 10.1038/s41443-026-01269-w

**Published:** 2026-04-23

**Authors:** Natalia Plamadeala, Wai Gin Don Lee, Antonio Ruffo, Mirko Preto, Angelo Di Giovanni, Parth Tagdiwala, Fabio Esposito, Paolo Gontero, David Ralph, Marco Falcone

**Affiliations:** 1 Department of Urology - A.O.U. “Città della Salute e della Scienza” – Molinette Hospital, University of Turin, Turin, Italy; 2 Department of Urology, University College London Hospitals NHS Foundation Trus, London, UK; 3 Department of Urology - Department of Medicine and Health Sciences “Vincenzo Tiberio”, University of Molise, Termoli, Italy; 4 Department of Urology, Casa di Cura Nostra Signora di Lourdes, Naples, Italy; 5 Neurourology Clinic - A.O.U. “Città della Salute e della Scienza” - Unità Spinale Unipolare, Turin, Italy; 6 Department of Urology, Biruni University, Istanbul, Turkey

**Keywords:** Surgery, Risk factors

## Abstract

Adult Acquired Buried Penis(AABP) is a debilitating condition often requiring surgical correction. This multicentre retrospective study, the largest of its kind in Europe to date, evaluated outcomes of 204 patients who underwent AABP repair between June 2011 and January 2025, with a median follow-up of 18.0 months(IQR 7.0–46.0). Surgical complexity was classified according to the Pariser system; 70.6% underwent high-complexity procedures(Pariser≥III). Body Mass Index ≥30 kg/m² was the leading etiological factor(45.1%), more prevalent in the high-complexity group(*p* < 0.001), followed by lichen sclerosus(23.0%), more prevalent in the low-complexity group(*p* = 0.011) and genital lymphoedema(20.1%). Main presenting complaints included sexual dysfunction(50.5%), aesthetic concerns(40.7%) and urinary problems(36.3%). Skin grafts were used in 44.6% of cases, more often in low-complexity procedures(*p* < 0.001). The overall complication rate was 27.0%, significantly higher in the high-complexity group(32.6% vs. 13.3%, *p* = 0.005), with high-complexity surgery independently predicting complications(*p* = 0.013). Recurrence occurred in 12.7%, with a recurrence-free survival rate of 91.5% and 83.7% at 12 and 24 months, respectively. Hematoma(*p* < 0.001) was associated with an increased risk of recurrence, whereas higher surgical complexity(*p* = 0.018) was associated with a reduced risk of recurrence. Postoperative gains included an increase in stretched penile length by 3.0 cm, improved urinary and sexual function(*p* < 0.001) and high satisfaction rates(86.8%). While high-complexity surgery leads to higher immediate complication rates, the comprehensive surgical approach, when tailored to the patient, offers durable results and significant improvements in function and quality of life.

## Introduction

Adult Acquired Buried Penis (AABP) is a morbid condition characterized by a normally sized phallus that becomes concealed beneath overhanging suprapubic fat, which, unlike the phallus anchored by the suspensory ligament, is unanchored and descends over the penis [1, 2]. The resulting moist environment promotes chronic inflammation, infection and progressive tissue entrapment [2, 3]. The condition has gained increasing attention over the past decade, paralleling the rising prevalence of obesity and metabolic syndrome, which are major contributing factors [4, 5]. Other causes include penoscrotal lymphedema, post-circumcision scarring, prior penile surgery (e.g. for cancer or bladder exstrophy) and genital lichen sclerosus (LS) [6–9].

Regardless of the cause, AABP may lead to urinary dysfunction, erectile dysfunction (ED) with painful erections, hygiene difficulties, recurrent infections and a higher risk of developing penile cancer [7, 10, 11], with substantial psychological distress and reduced quality of life (QoL) [12, 13]. In this context, surgical intervention becomes imperative when conservative treatments fail to manage the disorder. The primary goals are to re-expose the phallus, restore genital skin coverage, reconstruct genital teguments and eventually remove peri-genital or excess abdominal tissue to reduce recurrence and enhance QoL while minimising complications [14].

Multiple surgical approaches have been described. As categorized by Pariser et al., low-complexity procedures include unburial of the penile shaft, reconstruction of the penile shaft using skin flaps or grafts and plastic surgical techniques for scrotal reconstruction. High-complexity surgery incorporates excision of suprapubic fat (escutcheonectomy) and/or abdominal wall procedures such as apronectomy or abdominoplasty [15]. Despite advances, postoperative complications and recurrence remain clinically relevant and available evidence is frequently limited to single-centre series with heterogeneous case mix and reporting [16].

To better define contemporary outcomes and identify factors associated with recurrence and complications, we performed a multicentre cohort study across three high-volume referral centres using standardised data collection and a unified surgical complexity classification. We hypothesised that standardisation of the surgical approach across centres would improve surgical, functional and aesthetic outcomes while maintaining an acceptable rate of complications and recurrence.

## Materials and methods

We performed a multicentre retrospective cohort study across three referral centres for genitourinary reconstruction. Ethical approval was obtained at each participating centre and patients were consented according to local requirements.

Patients were eligible if they were ≥18 years old, diagnosed with AABP and underwent first-time AABP surgical repair between June 2011 and January 2025. A minimum follow-up of 6 months was required to allow assessment of early outcomes and recurrence. Patients with incomplete records or insufficient follow-up were excluded.

A standardised database template was used across all centres. Variables included demographics, comorbidities and risk factors, primary etiology of AABP, presenting complaints, operative details, postoperative course, complications, recurrence and patient-reported outcomes (PROs). Only the primary etiologic factor was recorded; other conditions were captured as comorbidities/risk factors and therefore percentages may differ across categories.

Procedures were classified according to the Pariser–Santucci system [15]. For procedures performed before its publication, operative reports were retrospectively reviewed and reclassified accordingly. Patients were stratified into low-complexity surgery (Pariser <III) and high-complexity surgery (Pariser ≥III). Patients undergoing multiple procedures were classified by the highest category performed. Surgical techniques have been previously described in our published work [17].

Postoperative management was tailored to the procedure. After abdominoplasty or suprapubic fat pad excision, bed rest with trunk flexion at approximately 30° was prescribed for 48 h. After penile shaft grafting, a compressive penile dressing and bladder catheter were maintained for one week. Scar optimisation included silicone-based and hydrating creams beginning approximately two weeks after surgery, alongside gentle scar massage.

Primary outcomes were recurrence-free survival (RFS) rate at 12 and 24 months and at last follow-up. Recurrence was defined as the reappearance of AABP at examination, with or without patient-reported symptoms, regardless of whether patients opted for further treatment. Secondary outcomes included predictors of recurrence and predictors of postoperative complications, as well as functional outcomes. Postoperative complications occurring within 3 months of surgery were classified according to the Clavien-Dindo system [18]. Functional outcomes were assessed preoperatively and at 12 months postoperatively and included: sexual function and lower urinary tract symptoms (LUTS) by using the validated International Index of Erectile Function (IIEF-15) [19] and the International Prostate Symptom Score (IPSS) [20], respectively. In addition, a custom 6-item PROs questionnaire (Supplementary Table) was administered at 12 months postoperatively.

Statistical analyses were conducted using the Statistical Package for the Social Sciences (SPSS; v. 29.0.2.0; IBM, Chicago, USA), with a two-sided significance level set at *p*-value ≤ 0.05. The normality of variable distributions was evaluated using the Kolmogorov–Smirnov test. Categorical variables are reported as frequencies and percentages. Continuous variables with a normal distribution are shown as means and standard deviations (SD), whereas those not normally distributed are presented as medians and interquartile ranges (IQR). Differences between groups were analysed using the Chi-square test or Fisher’s exact test for categorical variables or the Mann–Whitney test for continuous variables, as appropriate. The Log-Rank test, contingency tables and logistic regression were employed to examine the relationship between dependent and independent variables. The RFS rate was estimated through Kaplan-Meier analysis. Univariate and multivariable Cox regression analyses were conducted to report crude (cHR) and adjusted hazard ratios (aHR) in survival analysis. Confounding variables were defined as those known to be risk factors for the outcome. Variables with a *p*-value ≤ 0.20 in univariable Cox regression were considered for inclusion in the multivariable model. To prevent multicollinearity and overfitting, we excluded variables that were either conceptually redundant or had unstable estimates due to low event frequency, such as postoperative complications, particularly when overlapping with more specific events (e.g. hematoma) and complete graft loss. Missing data were excluded from specific analyses, with denominators adjusted accordingly.

## Results

### Study and patients’ characteristics

A total of 204 men underwent first-time AABP repair and met the inclusion criteria. Demographic and preoperative characteristics are summarised in Table 1. Median age was 54.0 years (IQR 41.0–64.8) and median follow-up was 18.0 months (IQR 7.0–46.0). High-complexity reconstruction was performed in 70.6% of cases. Median Body Mass Index (BMI) was 30.0 kg/m² (IQR 25.0–36.0). Prior bariatric surgery was present in 5.9% of patients, all within the high-complexity group (*p* = 0.020). A history of prior circumcision was reported in 37.3%.

**Table 1:** Baseline and preoperative characteristics of AABP patients.

Variables	Total (*n* = 204)	Low-Complexity (Santucci < III) (*n* = 60)	High-Complexity (Santucci ≥ III)(*n* = 144)	*p* Value
Nr of patients, *n* (%)	204 (100)	60 (29.4)	144 (70.6)	
Age, years (IQR)	54.0 (41.0–64.8)	60.0 (52.0–72.5)	52.5 (30.5–66.8)	0.563
Follow-up, months (IQR)	18.0 (7.0– 46.0)	19.5 (6.3–46.5)	16.0 (7.0–46.0)	0.376
Previous bariatric surgery, *n* (%)	12 (5.9)	0 (0)	12 (8.3)	0.020
Risk factors and comorbidities:				
• BMI, kg/m^2^ (IQR)	30.0 (25.0–36.0)	29.0 (23.9–39-0)	31.1 (28.3–35.0)	0.098
• BMI > 25 kg/m^2^, *n* (%)	137 (67.2)	31 (51.7)	106 (73.6)	0.003
• Obesity (BMI ≥ 30 kg/m^2^), *n* (%)	105 (51.5)	25 (41.7)	80 (55.6)	0.067
• OSAS, *n* (%)	16 (7.8)	3 (5.0)	13 (9.0)	0.404
• Smoke, *n* (%)	21 (10.3)	5 (8.3)	16 (11.1)	0.623
• Hypertension, *n* (%)	67 (32.8)	21 (35.0)	46 (31.9)	0.744
• Diabetes, *n* (%)	63 (30.9)	17 (28.3)	47 (32.6)	0.740
• Previous circumcision, *n* (%)	76 (37.3)	27 (45.0)	49 (34.0)	0.155
• Previous penile cancer surgery, *n* (%)	18 (8.8)	9 (15.0)	9 (6.25)	0.058

*AABP* adult acquired buried penis, *IQR* interquartile range, *BMI* body mass index, *OSAS* obstructive sleep apnea syndrome.

*P* values were calculated by the Mann–Whitney U test or Chi-square test or Fisher’s exact test.

Etiology and clinical presentation are shown in Table 2. The most common primary etiological factor was BMI ≥ 30 kg/m² (45.1%), followed by LS (23.0%) and genital lymphoedema/granuloma secondary to prior penile surgery or substance injections (20.1%). LS was more frequent in the low-complexity group than the high-complexity group (35.0% vs 18.1%, *p* = 0.011), whereas obesity was more common in the high-complexity group (54.9% vs 21.7%, *p* < 0.001). Presenting complaints included sexual dysfunction (50.5%), aesthetic concerns (40.7%), urinary symptoms (36.3%), poor hygiene (21.1%) and suspected penile cancer (1.0%), without statistically significant differences between groups.

**Table 2:** Etiology and clinical presentation of AABP patients.

Variables	Total (*n* = 204)	Low-Complexity (Santucci < III) (*n* = 60)	High-Complexity (Santucci ≥ III)(*n* = 144)	*p* Value
Nr of patients, *n* (%)	204 (100)	60 (29.4)	144 (70.6)	
Etiology, *n* (%)				
• Lichen Sclerosus	47 (23.0)	21 (35.0)	26 (18.1)	0.011
• Obesity (BMI ≥ 30 kg/m^2^)	92 (45.1)	13 (21.7)	79 (54.9)	<0.001
• Previous circumcision	11 (5.4)	6 (10.0)	5 (3.5)	0.086
• Genital lymphedema/granuloma	41 (20.1)	15 (25.0)	26 (18.1)	0.258
• Previous penile surgery	13 (6.4)	5 (8.3)	8 (5.6)	0.531
Patients’ complaints, *n* (%)				
• Poor hygiene	43 (21.1)	13 (21.7)	30 (20.8)	1.000
• Urinary issue	74 (36.3)	20 (33.3)	54 (37.5)	0.633
• Sexual issue	103 (50.5)	30 (50.0)	73 (50.7)	1.000
• Aesthetic issue	83 (40.7)	21 (35.0)	62 (43.1)	0.348
• Suspected penile cancer	2 (1.0)	2 (3.3)	0 (0)	0.085

*AABP* adult acquired buried penis, *IQR* interquartile range, *BMI* body mass index.

*P* values were calculated by the Mann–Whitney U test or Chi-square test or Fisher’s exact test.

### Surgical outcomes

Operative details and surgical outcomes are summarised in Table 3. Median hospital stay was 3.0 days (IQR 2.0–5.0) and median operative time was 120 minutes (IQR 95.0–150.0).

**Table 3:** Surgical characteristics and outcomes of AABP repair.

Variables	Total (*n* = 204)	Low-Complexity (Santucci < III) (*n* = 60)	High-Complexity (Santucci ≥ III) (*n* = 144)	*p* Value
Hospital stay (days), (IQR)	3.0 (2.0–5.0)	2.0 (1.0–4.0)	3.0 (2.0–5.0)	0.029*
Operation time (min), (IQR)	120 (95.0–150.0)	115.0 (65.0–150.0)	120.0 (90.3–150.0)	0.056*
Pariser–Santucci classification, *n* (%)				
• Category I	8 (3.9)			
• Category II	53 (26.0)			
• Category III	12 (5.9)			
• Category IV	59 (28.9)			
• Category V	72 (35.3)			
Use of skin graft, *n* (%)	91 (44.6)	40 (66.7)	51 (14.6)	<0.001*
Type of skin graft, *n* (%)				
• FTSG	35 (38.5)	14 (23.3)	21 (14.6)	0.665*
• STSG	56 (61.5)	26 (43.3)	30 (20.8)	0.665*
Postoperative complications, nr. patients (%)	55 (27.0)	8 (13.3)	47 (32.6)	0.005*
Clavien classification, *n* (%)				
• I	30 (14.7)	5 (8.3)	25 (17.4)	
• II	20 (9.8)	2 (3.3)	18 (12.5)	
• ≥III				0.499*
IIIa	4 (2.0)	1 (1.7)	3 (2.1)	
IIIb	1 (0.5)	0 (0)	1 (0.7)	
Types of complications, n (%)				
• Wound infection	13 (6.4)	0 (0)	13 (9.0)	0.011*
• Hematoma	18 (8.8)	1 (1.7)	17 (11.8)	0.016*
• Oedema	8 (3.9)	2 (3.3)	6 (4.2)	1.000*
• Wound breakdown	27 (13.2)	4 (6.7)	23 (16.0)	0.074*
• Lymphedema	4 (2.0)	1 (1.7)	3 (2.1)	1.000*
• UTI	4 (2.0)	1 (1.7)	3 (2.1)	1.000*
Complete graft loss, *n* (IQR)	2 (1.0)	1 (1.7)	1 (0.7)	1.000*
Partial graft loss, *n* (IQR)	8 (3.9)	2 (3.3)	6 (4.2)	0.237*
Presence of LS at histology, *n* (%)	29 (14.2)	11 (18.3)	18 (12.5)	0.649*
Buried penis recurrence at follow-up, *n* (%)	26 (12.7)	11 (18.3)	15 (10.4)	0.165*
RFS rate at 12 months, %	91.5	91.5	91.6	0.952^α^
RFS rate at 24 months, %	83.7	78.2	86.5	0.304^α^
Preop penile length, cm (IQR)	7.0 (5.0–9.0)^β^	7.0 (4.3–10.5)^β^	6.5 (5.0–9.0)^β^	0.501*
Post penile length, cm (IQR)	11.0 (8.9–13.0)^β^	12.0 (8.8–13.0)^β^	11.0 (8.6–12.0)^β^	0.616*
Difference in penile length, cm (IQR)	3.0 (2.0–6.0)	2.5 (1.8–6.0)	3.3 (1.0–6.0)	0.814*

*AABP* adult acquired buried penis, *IQR* interquartile range, *FTSG* full thickness skin graft, *STSG* split-thickness skin graft, *UTI* urinary tract infection, *RFS* recurrence-free survival.

* Mann–Whitney U test or Chi-square test or Fisher’s exact test.

^α^ Log-Rank test.

^β^ all pre- and post-surgery comparisons were performed using the Wilcoxon signed-rank test, with *p*-values < 0.001.

Skin grafting was performed in 44.6% of cases and was more frequent in the low-complexity group (66.7% vs 14.6%, *p* < 0.001). Among grafts, split-thickness skin grafts (STSG) were used more commonly than full-thickness grafts (FTSG).

**Fig. 1:**
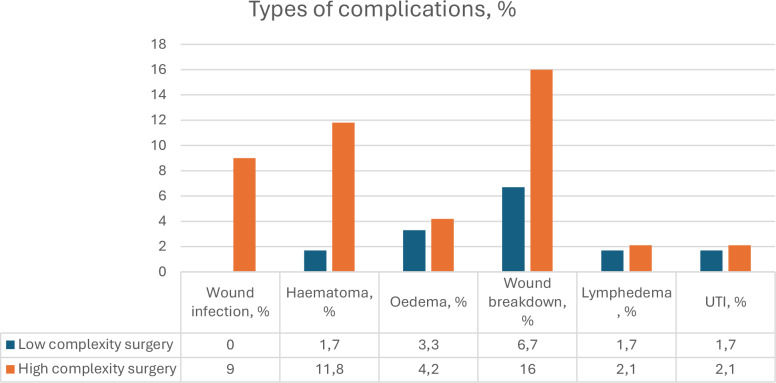
Overall postoperative complications according to the type of surgery.

Postoperative complications occurred in 55 patients (27.0%) and were more frequent after high-complexity surgery (32.6% vs 13.3%, *p* = 0.005). In total, 74 complications were recorded, as some patients experienced more than one event (Fig. 1). Hematoma formation (*p* = 0.016) and wound infection (*p* = 0.011) were significantly more frequent in the high-complexity group. Most complications were minor (Clavien <III). Major complications requiring surgical revision (Clavien ≥III) occurred in 2.5%, predominantly following high-complexity surgery, including two cases of genital wound breakdown with complete STSG loss, one infection with tissue breakdown (requiring debridement) and two hematomas requiring surgical evacuation. No Clavien IV or V events occurred.

Stretched penile length increased significantly from pre- to postoperative assessment (*p* < 0.001) with no difference between complexity groups. Overall median length increased from 7.0 cm (IQR 5.0–9.0) to 11.0 cm (IQR 8.9–13.0), corresponding to a median gain of 3.0 cm (IQR 2.0–6.0).

### Recurrence and RFS rate

**Fig. 2:**
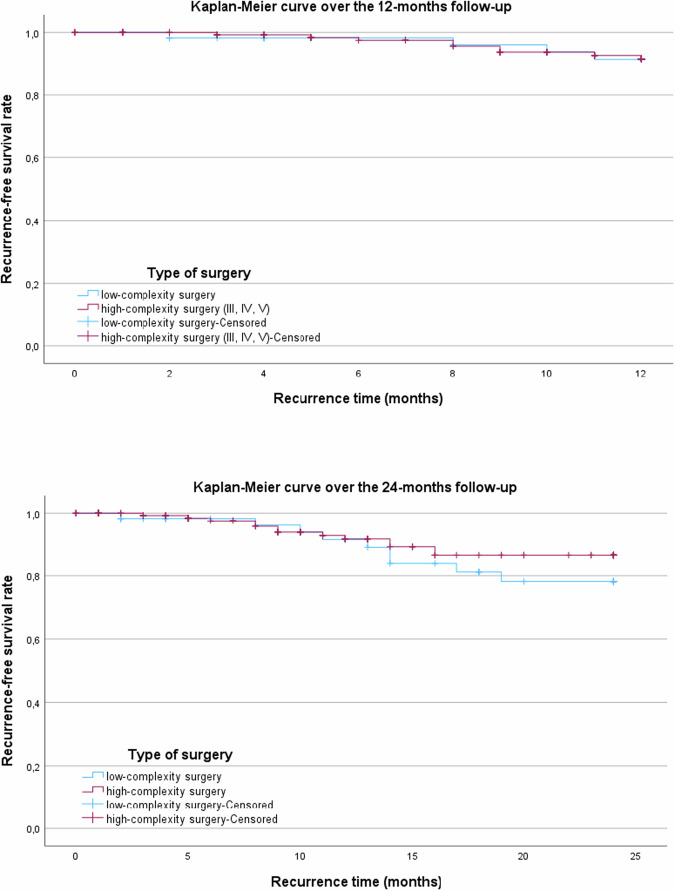
Kaplan–Meier curves of Recurrence Free Survival rate at 12- and 24-month follow-up in patients undergoing low- versus high-complexity AABP repair.

Overall, 26 patients (12.7%) developed recurrence during follow-up. RFS rate was 91.5% at 12 months and 83.7% at 24 months. Kaplan–Meier analysis showed no significant difference between complexity groups at 12 months (log-rank = 0.952) or 24 months (log-rank = 0.304) (Fig. 2).

Among patients with recurrence, the median time to recurrence was 12.5 months (IQR 8.0–16.3) with no difference between complexity groups (*p* = 0.532).

### Functional outcomes and PROs

A total of 68 patients (33.3%) completed functional questionnaires (Tables 4–5). Responders were similar to the overall cohort in key demographic, clinical and surgical characteristics; however, non-response bias cannot be fully excluded.

**Table 4:** Functional outcomes (68 patients) of AABP repair: urinary and sexual functions.

Variables	Total (*n* = 68)	Low-Complexity (Santucci < III) (*n* = 16)	High-Complexity (Santucci ≥ III) (*n* = 52)	*p* Value
IPSS pre-surgery, (IQR)	12.0 (5.0–20.0)	11.0 (5.5–14–0)	12.5 (4.8–20.5)	0.563
IPSS post-surgery, (IQR)	2.0 (0.0–8.0)	3.0 (0.0–10.5)	2.0 (0.0–7.3)	0.490
IIEF 15 pre-surgery, (IQR)	27.0 (15.0–65.0)	25.0 (12.0–66.5)	29.5 (15.0–65.0)	0.368
IIEF 15 post-surgery, (IQR)	51.0 (16.0–70.0)	62.0 (13.0–70.0)	46.5 (16.8–70.3)	0.880

*AABP* adult acquired buried penis, *IQR* interquartile range, *IPSS* international prostate symptom scrore, *IIEF* international index of erectile function.

All *p* values were calculated by Mann–Whitney U test or Wilcoxon signed-rank.

All comparisons pre- and post-surgery are *p* < 0.001.

**Table 5:** Patient-reported outcomes (68 patients) following AABP repair.

Variables	Total (*n* = 68)	Low-Complexity (Santucci < III) (*n* = 16)	High-Complexity (Santucci ≥ III) (*n* = 52)	*p* Value
Overall satisfaction for the operation, *n* (%)	59 (86.8)	15 (93.8)	44 (84.6)	0.224
Overall improvement of urinary function, *n* (%)	56 (82.4)	15 (93.8)	41 (78.8)	0.333
Overall improvement of genital hygienic care, *n* (%)	58 (85.3)	14 (87.5)	44 (84.6)	1.000
Would you suggest a friend undergo the same operation? *n* (%)	60 (88.2)	13 (81.3)	47 (90.4)	0.380
Improvement of sexual life after surgery, *n* (%)	60 (88.2)	14 (87.5)	44 (84.6)	1.000
Positive impact of surgery on quality of life, *n* (%)	59 (86.8)	14 (87.5)	45 (86.5)	1.000

*AABP* adult acquired buried penis.

All *p* values were calculated by chi-square test.

Both IPSS and IIEF-15 significantly improved at 12 months postoperatively (*p* < 0.001), with no differences between low- and high-complexity procedures. Across all PRO domains, over 78.8% of patients reported improvement. Overall satisfaction was 86.8% and improvement in QoL was 86.8%.

### Predictors of AABP recurrence

In the multivariate Cox regression model, after adjustment for potential confounding variables (with *p* ≤ 0.2 in crude analysis), hematoma (aHR 6.64, 95% CI 2.5–18.0, *p* < 0.001) emerged as an independent risk factor for recurrence, while high-complexity reconstruction (aHR 0.35, 95% CI 0.1–0.8, *p* = 0.018) was independently associated with a reduced risk, acting as a protective factor. Other variables, such as BMI ≥ 30 kg/m^2^, prior circumcision and previous bariatric surgery, did not retain statistical significance after adjustment. The loss of significance for obesity-related variables (BMI ≥ 30 kg/m^2^ and prior bariatric surgery) in the adjusted model suggests that their association with recurrence may be attributed to an increased risk of postoperative complications or impaired wound healing.

However, in logistic regression, higher BMI and previous circumcision were significantly associated with increased risk of recurrence (odds ratio OR, 1.06, 95% CI 1.01–1.12, *p* = 0.032 and OR 3.00, 95% CI 1.28–7.05, *p* = 0.012, respectively). Each 1-unit increase in BMI was associated with a 6% higher risk of BP recurrence. These associations did not remain significant in the Cox regression model, suggesting that BMI and previous circumcision may influence the occurrence, but not the timing of recurrence. (Table 6)

**Table 6:** Univariate and Multivariate analysis of risk factors for recurrence in patients who underwent AABP surgical repair.

	Univariate analysis		Multivariate analysis	
	cHR (95% CI)	*p* for cHR	aHR (95% CI)	*p* for aHR
Previous circumcision	2.6 (1.2–5.8)	0.016	1.83 (0.8–4.4)	0.179
BMI ≥ 30 kg/m^2^	2.2 (0.9–5.0)	0.068	1.97 (0.8–5.0)	0.156
LS at histopathology	1.1 (0.4–2.8)	0.910		
Previous bariatric surgery	3.8 (1.1–12.8)	0.032	2.85 (0.8–10.9)	0.126
High complexity surgery	0.6 (0.3–1.3)	0.200	0.35 (0.1–0.8)	0.018
Complications < 90 days	2.8 (1.3–6–0)	0.010		
− Genital wound infection	2.1 (0.6–7.0)	0.231		
− Hematoma	5.2 (2.2–12.5)	<0.001	6.64 (2.5–18.0)	<0.001
− Wound breakdown	1.3 (0.5–3.5)	0.571		
− Lymphedema	1.7 (0.2–12.7)	0.600		
− UTI	2.8 (0.4—20.5)	0.319		
Clavien ≥ III	1.8 (0.4–8.3)	0.446		
Graft complete loss	4.0 (0.5–30.8)	0.186		

*AABP* adult acquired buried penis, *BMI* body mass index, *LS* Lichen Sclerosus, *UTI* urinary tract infection, *CI* confidence interval, *cHR* crude Hazard ratio, *aHR* adjusted Hazard ratio.

All *p*-values were calculated using univariable and multivariable Cox regression analyses.

### Predictors of postoperative complications

In multivariable logistic regression, high-complexity reconstruction was independently associated with increased risk of postoperative complications (OR 2.89, 95% CI 1.25–6.68, *p* = 0.013). No other variables—including age, BMI, obstructive sleep apnoea syndrome, hypertension, diabetes or current smoking—were significantly associated with complications (all *p* > 0.05).

## Discussion

This multicentre cohort represents the largest European dataset to date evaluating surgical reconstruction for AABP and provides contemporary evidence regarding surgical outcomes, functional recovery and predictors of recurrence and complications. In 204 men managed across three high-volume centres, AABP repair achieved high RFS rates, meaningful anatomical restoration, significant improvements in urinary and sexual function and high patient satisfaction.

Consistent with previous reports, a substantial proportion of patients (51.5%) in our cohort had a BMI ≥ 30 kg/m^2^, supporting the role of excess suprapubic adiposity in the pathogenesis of AABP [13, 16, 17, 21]. The condition predominantly affected middle-aged men, with a median age of 54.0 years, which overlaps with the peak incidence of penile cancer in the sixth decade of life. Because AABP shares several risk factors with penile cancer [22] and may impair genital hygiene and physical examination, delayed recognition of premalignant and malignant lesions has been reported in up to 35.0% and 7.0–9.1% of cases, respectively [7, 10, 23]. These data reinforce the importance of timely diagnosis and referral for definitive management.

From a reconstructive perspective, most grafted patients in our series received STSGs (61.5% out of 91 patients), with greater use in low-complexity procedures. AABP frequently compromises genital and perigenital skin quality and the coexistence of genital LS—reported in 10.6% to 62.7% of cases—further limits the availability of healthy local tissue. Consequently, FTSGs harvested from genital areas are generally discouraged, as diseased or poor-quality skin may impair graft take, compromise durability and increase recurrence risk [23–25].

Standardised classification using the Pariser–Santucci system [15] allowed consistent reporting across centres and facilitated comparison of surgical outcomes. High-complexity reconstruction was required in 70.6% of patients, consistent with previously reported rates ranging from 69.0% to 76.1% [15, 17]. Although associated with higher early complication rates, these procedures achieved comparable RFS rates and significant functional improvement. These findings support an individualized surgical strategy tailored to disease extent rather than a uniform procedural approach.

Overall, recurrence occurred in 26 of 204 patients (12.7%), corresponding to RFS rates of 91.5% at 12 months and 83.7% at 24 months, with a median time to recurrence of 12.5 months. These outcomes align with previously reported success rates ranging from 85% to 100% [2, 15, 17, 26, 27]. As most recurrences occurred within the first two years, regular postoperative surveillance during this period is strongly recommended.

Unlike the findings of Pariser et al. [15], who reported a higher RFS rate in the low-complexity group compared to the high-complexity group (100% vs. 86%), our cohort showed no statistically significant differences in RFS rates between surgical groups. This may reflect differences in patient characteristics (e.g. BMI, comorbidities, AABP etiology) or subtle variations in surgical and perioperative practices. Notably, all cited studies, including ours, originate from high-volume centres, yet these findings highlight the need for particular attention in lower-volume settings, where outcomes may be more variable.

Our dataset identified two key predictors of recurrence following surgical repair of AABP. Postoperative hematoma was independently associated with a 6.64-fold higher risk of recurrence, suggesting that postoperative bleeding may adversely affect outcomes through increased inflammation, impaired wound healing or reduced graft take. Conversely, high-complexity reconstruction was associated with an approximately 65% reduction in recurrence risk, potentially reflecting a more definitive correction in appropriately selected patients.

However, in the absence of a standardised measure of baseline AABP severity, surgical complexity was used as a surrogate for disease severity and may not fully capture the extent of preoperative disease. Consequently, some patients managed with low-complexity procedures may have had more advanced disease, potentially resulting in undertreatment and contributing to higher recurrence rates in this group. Therefore, the association between surgical complexity and recurrence should be interpreted with caution.

Together, these findings underscore the importance of meticulous intraoperative hemostasis and suggest that more comprehensive reconstructive approaches, when appropriately indicated, may improve long-term outcomes.

Although BMI was not identified as a predictor of recurrence in our cohort, previous findings by Chestnut et al. [28] reported a strong association between elevated BMI and recurrence risk, including a 12.7-fold increase in recurrence for BMI ≥ 40 kg/m² and a 12% increase in risk for each additional BMI unit, supporting the role of obesity as a potentially modifiable factor in preoperative assessment and counselling.

The overall complication rate was 27.0%, consistent with previously reported ranges, although some series have described rates as high as 80.8% [15, 21, 29–31]. High-complexity reconstruction was independently associated with increased postoperative morbidity (OR 2.89, 95% CI 1.25–6.68, *p* = 0.013), reflecting the extent of surgical intervention required in advanced disease. Most complications were minor and managed conservatively, with only 2.5% of patients requiring surgical revision for Clavien ≥ III events. This finding is consistent with previous studies: Cooper et al. [32] identified abdominal panniculectomy as a risk factor for complications and Aubé et al. [31] reported that it significantly increased the risk of major (Clavien ≥ III) complications (HR 28).

While BMI was not independently associated with complications in this cohort, prior studies report increasing BMI as a risk factor for morbidity and recurrence, indicating that obesity remains clinically relevant for counselling and optimisation even when it does not retain independent significance in adjusted models. Aubé et al. [31] found that a BMI ≥ 40 kg/m^2^ and active smoking were strong independent predictors of overall complications (HR 25 and 14.6, respectively). Hampson et al. [26] similarly observed a linear relationship between increasing BMI and complication risk. Chestnut et al. [28] confirmed this association, showing that a BMI ≥ 38 kg/m² was associated with a 6.7-fold increase in the odds of postoperative complications (*p* = 0.020), with each additional BMI point increasing risk by 11%.

Anatomical restoration was accompanied by meaningful functional improvement. The median increase in stretched penile length was 3.0 cm, consistent with prior literature, ranging from 2.0 to 3.8 cm [17, 33–35].

This anatomical improvement is accompanied by a functional benefit, addressing the primary complaints that led patients to seek consultation. In our cohort, both urinary and sexual functions significantly improved postoperatively. Specifically, the IPSS score decreased by 10 points, indicating better urinary flow and reduced lower urinary tract symptoms, while the IIEF-15 score increased by 34 points, reflecting enhanced sexual function. Notably, no statistically significant differences were observed between surgical groups, suggesting that functional recovery is achievable across varying levels of surgical complexity when procedures are properly tailored to the patient. Overall, patient-reported satisfaction and perceived improvement in quality of life were high, both reaching 86.8%, in line with current literature [11–13, 21, 26].

### Limitations

Although, to our knowledge, this study presents the largest European cohort of patients undergoing surgical repair for AABP, several limitations must be acknowledged. The retrospective nature of data collection limits the ability to draw definitive conclusions regarding certain outcomes. Additionally, not all patients completed the functional questionnaires, introducing potential non-response bias. Functional questionnaires were completed by one-third of the cohort and although responders were broadly representative, non-response bias cannot be excluded. The IPSS and IIEF-15, although validated in English, have not been formally validated in Italian, potentially affecting the accuracy of functional assessment. The long inclusion period and multicentre design introduce potential heterogeneity in operative technique and surgeon experience, although standardised classification was applied to improve consistency. The lack of randomisation is another limitation, although it is not feasible in this clinical setting. Future research should focus on establishing a multicentre prospective registry with standardised data collection and consistent use of validated PROMs to improve data completeness, comparability and external validity of findings.

## Conclusion

The management of AABP remains a complex and evolving challenge within genitourinary and abdominal reconstructive surgery. Our findings demonstrate that surgical repair offers high RFS rates, a median gain of 3 cm in stretched penile length and significant improvements in urinary function, sexual satisfaction and overall quality of life. Although high-complexity procedures are associated with a higher rate of early postoperative complications, a patient-tailored and comprehensive surgical approach provides durable functional and quality-of-life benefits. These results highlight the importance of meticulous surgical technique, optimized perioperative management and careful patient selection. Early referral to specialized, high-volume centres—where multidisciplinary expertise and standardised protocols are available—may further reduce complications and enhance long-term outcomes. Future prospective multicentre registries incorporating standardised data and validated PROMs are warranted to refine surgical strategies and establish evidence-based standards of care.

## Supplementary information

AABP secondary to sclerosing granuloma

AABP secondary to partial penectomy

AABP secondary to obesity

Ad ‘hoc’ 6-items questionnaire

## Data Availability

The data that support the findings of this study are available from the corresponding author (SW) upon reasonable request.
