# Supplementary figures and images for: Outcomes of adult acquired buried penis (AABP) reconstruction: a multicentre cohort study
Source: Int J Impot Res. 2026 Apr 23;38(4):354–62. doi: 10.1038/s41443-026-01269-w (PMC13132718; doi:10.1038/s41443-026-01269-w)

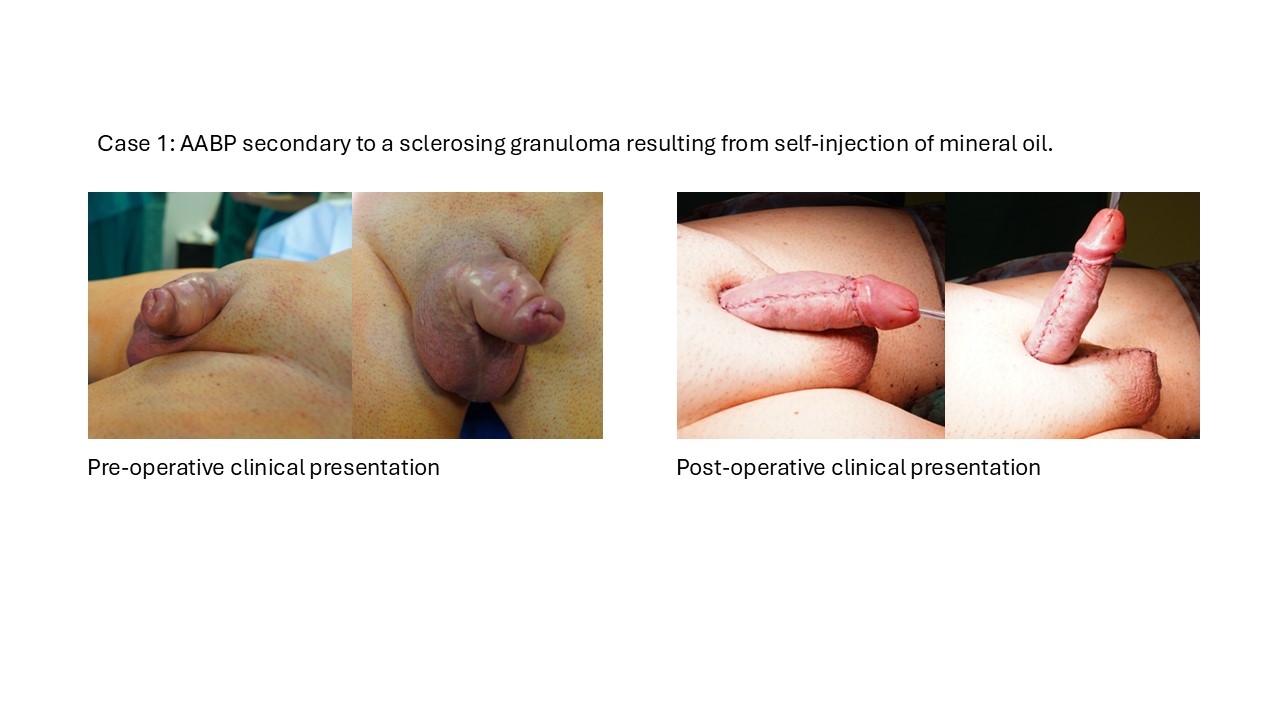

Supplement: Supplementary file 1 — AABP secondary to sclerosing granuloma [file 41443_2026_1269_MOESM1_ESM.jpg]

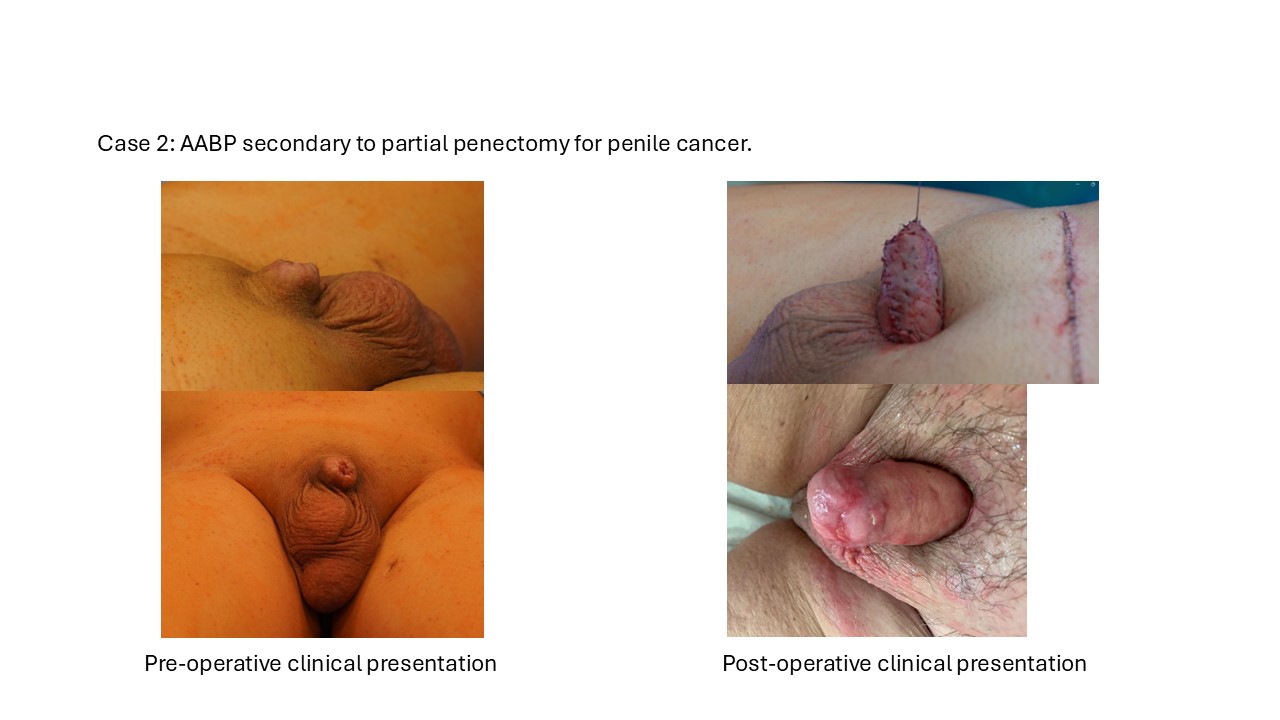

Supplement: Supplementary file 2 — AABP secondary to partial penectomy [file 41443_2026_1269_MOESM2_ESM.jpg]

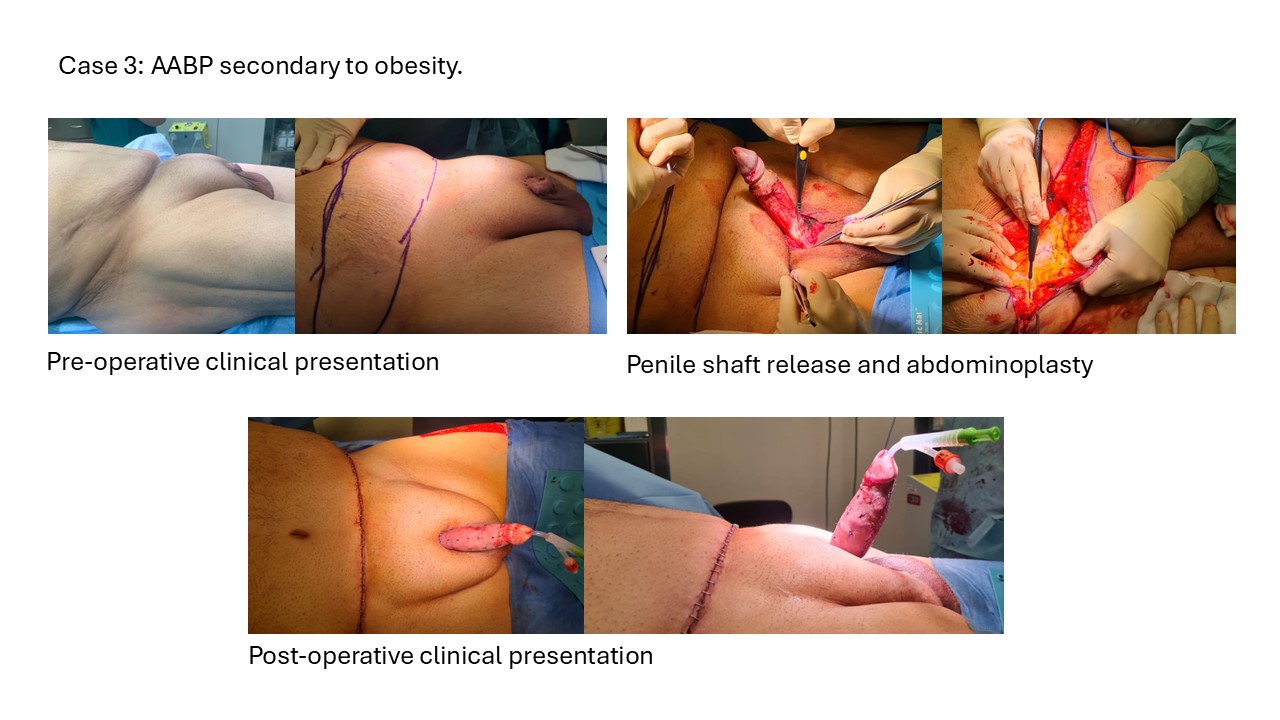

Supplement: Supplementary file 3 — AABP secondary to obesity [file 41443_2026_1269_MOESM3_ESM.jpg]
