# Supplementary material for: Outcomes of adult acquired buried penis (AABP) reconstruction: a multicentre cohort study
Source: Int J Impot Res. 2026 Apr 23;38(4):354–62. doi: 10.1038/s41443-026-01269-w (PMC13132718; doi:10.1038/s41443-026-01269-w)
Supplement: Supplementary file 4 — Ad ‘hoc’ 6-items questionnaire [file 41443_2026_1269_MOESM4_ESM.pdf]

Ad “hoc” 6-items questionnaire

	YES	NO
1. Overall, are you satisfied with the intervention?		
2. Did the surgery have a positive impact on the quality of your urinary function?		
3. Did the surgery improve your genital hygiene?		
4. Would you recommend the surgery to a friend/family member?		
5. Did the surgery improve your quality of life?		
6. Did the surgery have a positive impact on the quality of your sexual life?		
